# Retraction: Zoledronic Acid Restores Doxorubicin Chemosensitivity and Immunogenic Cell Death in Multidrug-Resistant Human Cancer Cells

**DOI:** 10.1371/journal.pone.0244194

**Published:** 2020-12-15

**Authors:** The PLOS ONE Editors

Following the publication of this article [1], concerns were raised regarding results reported in Figs 2, 3, 4, 5, and 6. Specifically:

- Irregularities including straight horizontal and vertical distortions of the background and/or repetitive elements in the background noise were detected in the panels listed below:
  ○ Fig 2D, GAPDH panel
  ○ Fig 3A, pHIF-1α panel and HIF-1α panel
  ○ Fig 3C, *pro mdr*1 panel
  ○ Fig 3D, GAPDH panel
  ○ Fig 4A, HIF-1α panel
  ○ Fig 4C, Pgp panel
- In Fig 3C, the first three lanes of the Input panel representing HT29 CTRL, HT29 ZA, and HT29-dx CTL appear similar to lanes 4–6 representing HT29-dx ZA, HMM CTRL, and HMM ZA respectively.
- In Fig 5B, the following Ponceau staining results appear similar:
  ○ HT29 CTRL, HT29 ZA, HT29-dx CTRL, HT29-dx ZA, HMM CTRL, and HMM ZA
  ○ HT29 Dox, HT29-dx Dox, and HMM Dox
  ○ HT29 Dox+ZA, HT29-dx Dox+ZA, and HMM Dox+ZA
- The FACS panels representing DC ^DX CTRL^ and DC ^DX ZA^ in Fig 6C appear similar.
- In Fig 6C, the FACS results presented for DC ^DX Dox^ appear more similar to the FACS results for DC ^DX Dox+ZA^ than would be expected from independent FACS samples.

The authors have provided higher resolution images of the published panels but indicated that the original data underlying the published results are no longer available. The data provided were not sufficient to resolve the concerns summarised above and raised further concerns pertaining the integrity of the blots presented in this article.

In light of the concerns affecting multiple figure panels that question the integrity of these data, the *PLOS ONE* Editors retract this article.

CR, BC, JK, MC, AB, and MM did not agree with the retraction. IC, GP, and DG either could not be reached or did not respond directly.
